# Life Perspective and Social Health after Acid Burn: An Observational Study of Three Victims

**DOI:** 10.7759/cureus.9546

**Published:** 2020-08-04

**Authors:** Unsa Athar, Saad Ur Rahman, Seemab Imtiaz Gill, Taimoor Jamil, Muhammad Awais Sharif, Muhammad Khawar Sana

**Affiliations:** 1 Community Medicine and Public Health, Shalamar Medical and Dental College, Lahore, PAK; 2 Community Medicine, King Edward Medical University, Lahore, PAK; 3 Internal Medicine, King Edward Medical University, Lahore, PAK

**Keywords:** acid attack victims, vitriolage, social life, life perspective, trauma

## Abstract

After an acid attack, also known as vitriolage, many patients suffer from changes in life perspective, behavior, feelings, social withdrawal, social isolation, and depression. Formal and informal social support is vital for the proper and complete rehabilitation of acid burn victims. The government should form separate public help centers for such patients. The need of the hour, however, is the invention of proper legislation for the prevention of this heinous crime.

## Introduction

Social scientists all over the world are focusing on violence against women in developing and developed countries [1]. The majority of acid burns occur in developing countries where the ability to treat and provide for this population is severely limited due to limited resources for surgery and rehabilitation [2]. Violence against women was defined in the United Nations (UN) Declaration on the Elimination of Violence Against Women. The definition, adopted by the General Assembly in 1993, has described violence against women as; “any act of gender-based violence that results in, or is likely to result in, physical, sexual, or psychological harm or suffering to women, including threats of such acts, coercion or arbitrary deprivation of liberty, whether occurring in public or private life” [3]. Acid throwing, or vitriolage, is being considered a worth-discussing concern in Pakistan nowadays [4].

According to a research done in Bangladesh, acid is used as a common tool for violence because it is cheap and easily accessible [5]. Research from Cambodia showed that acid throwing does not have medical effects only, it damages the psychological, social, and economic aspects of a victim’s life as well [6].

Our study aimed to find out about the change in life perspective of acid burn victims after the incident and evaluate the effects on their personal and social life. We planned to investigate the following: (a) consequences of acid burn violence on women’s social health, (b) identify and understand the context associated with life perspective and feelings of an acid burn victim, and (c) identify the formal and informal support offered to an acid burn victim. Our study will help to formulate ethical and legislative rules and regulations to rehabilitate acid burn victims and to prevent such accidents from happening.

## Materials and methods

This is an observational study of three cases. Particpant #1 is A, an unmarried woman and participant #2 is B, her young sister, who suffered acid burn violence. Particpant #3 is C, a married factory worker from the urban slums of Lahore. All of them are of poor socio-economic status residing in an urban area. The victims were identified from the records of acid burn patients presenting to Mayo hospitals, Lahore, Pakistan in past years. They were called to follow-up their case. The respondents were assured of the confidentiality and informed consent was obtained but the use of audiotape machines, cellular phones, and taking photographs was not allowed. However, the research assistant was allowed to take notes.

Two sessions were held with each participant. The first was to develop the rapport while the second was an in-depth interview. Data were collected using a question guide.

The research assistant took notes of the interview, which were later tallied with the observations. The second interview was conducted to fill out the gaps in the previous one. The content analysis is done by appropriate consistent coding.

## Results

The victim, A, aged 20 years can speak two languages Urdu and Punjabi. She is an illiterate woman who lived with her parents, with her two elder brothers and one younger sister. Her sister is victim #2, B. Her sister and she both experienced acid burn, and psychological harassment as a result. The acid was thrown on her face and neck by her neighbor in the middle of the night. Her sister, B, aged 6, was sleeping next to her, who also suffered an acid burn on her right arm and chest. The neighbor was a male, who threw acid on her face after she turned down the proposal from him. She had partial blindness, partial deafness, and severe disfigurement. The victim suffered from psychological assault from the relatives and neighbors after the incident, which led to their family moving to another neighborhood. The younger girl has no permanent scarring but is psychologically traumatized.

The third victim, C, is a married, 30-year-old lady who used to work at a factory in an urban slum. She has two children. She got in an argument with the factory manager over the delay in dispersing salaries. She threatened to file an official complaint in the police. The next day, the manager threw acid on her face and said it was an accident. She was fired from her job. C feels socially isolated and depressed. Her husband is unemployed and blames her for losing her job. She feeds her children with help from her parents, who also brought her to the Mayo Hospital for treatment and management.

Theme #1 - life perspective, behavior, and feelings

The main theme that came up after the interview was a change in the women’s perspective about life. The younger girl did not understand fully the concept of life and behavior but seemed less talkative and cheerful than a child of her age should be. The elder sister was doing all the talking for her sister as well.

The major theme of life perspective can be described into three sub-themes; depression/inferiority complex, anger, and hope (Table 1).

**Table 1:** Theme #1 - life perspective, behavior, and feelings

Sub-theme# 1.1	Depression/Inferiority Complex
A	“Why didn’t God stop that horrible man from doing that to me? Was it all my fault? That I said NO to him? I shouldn’t have said No. I want this scar to go away. I cannot show my face to anyone. My family suffered because of me. What was their fault? What was my sister’s fault? Why was she burnt?”
B	“I am like appa (elder sister). I am ugly. No children want to play with me anymore.”
C	“Why do you want to see my scar? Can you treat it? If no, you shouldn’t see it. It’s meant to be hidden.”

Sub-theme# 1.2	Anger
A	“They say there is no proof of what he did. Is my face not proof enough? I wish I could strangle him. Or throw acid on his face. To make him suffer the same pain.”
B	“The man did not receive punishment. Why?”
C	“I want to scream and cry. I get so mad that I want to beat him to death with a bat.”

Sub-theme# 1.3	Hope
A	“I need to keep trying to get my scars removed. I was beautiful before this. I had never realized the beauty God gave me. I pray to him every day now to give me back my beauty. God will help me find a life partner.”
B	“Mama says God listens to children’s prayers faster. I pray that He makes me and appa perfect again?”
C	“I often wonder how have I survived this pain until now. I think my children give me the strength to live. They are the reason I am putting up with my horrible husband. They make me believe in the justice of God, they are my angels”

Theme #2 - social stigmatization and social withdrawal

The victims suffered from social stigmatization and social withdrawal because of the permanent scarring of their faces and bodies. There was a feeling of self-blame which led to further social isolation. Seeing the pity or disgust in people’s eyes made the victims recoil which led to limited social interactions. Constant victim shaming and body shaming also contributed towards the social withdrawal. Victim shaming and social isolation were the two sub themes seen, as described in Table 2.

**Table 2:** Theme #2 - social stigmatization and social withdrawal

Sub-theme# 2.1	Victim shaming
A	“Everyone in the neighborhood pointed at me and talked about me. They thought it was my fault. Because I am a girl. I cannot live with my relatives. They treat me like a useless animal. They think I cannot get married now so I am of no use. Everyone says it’s my fault that mother and father both had heart issues after the incident. I ruined my parents with stress.”
B	“Everyone tells me it was appa’s fault that I am ugly now. They say I should hate her.”
C	“My husband blames me for all that happened. He says that I should have known better than to argue with my boss. He says it is my fault that my children are hungry. He wants me to find a job because he cannot work.”

Sub-theme# 2.2	Social Isolation
A	“Everybody looks at me with pity in their eyes. They say what a shame, she lost her prettiness. I hate that. I do not want to show my face to anyone ever again. My parents cannot force someone to marry me. I was supposed to get married and have kids and serve my husband, like my mom did all her life. What am I supposed to do now? Wait for a miracle?”
B	“No one wants to play with me anymore. They say I am cursed. I need to make new friends but I cannot.”
C	“Everyone has abandoned me. My parents help but for how long will they keep helping? I have no friends or neighbors I can talk to. My life partner is of no use as well. I feel so alone.”

Theme #3 - social support

Formal

The public sector hospitals (Gujranwala Hospital and Mayo Hospital) funded by the government were involved in management and rehabilitation after abuse. The victims underwent many reconstructive surgeries. They did try to contact non-government organizations but it was hard to find one without contacts. The caregivers helped them to the best of their abilities regarding their health problems and physical disabilities, but could not play a substantial role in their rehabilitation. The formal social support must be empowered and funded to rehabilitate acid burn victims (Table 3).

Informal

The victim received strong informal social support. Their parents, children, and siblings provided informal social support like emotional, moral, and financial support without any pre-defined criteria or policies. Their families took them to hospitals to get the surgeries done. The victims seemed grateful and indebted to the family for all the informal support they provided (Table 3).

**Table 3:** Theme #3 - social support

Subtheme# 3.1	Formal Support
A	“All these NGOs and TV channels, they need pictures and videos to show off. No one helps you if they do not have anything to gain out of it. The government hospital has been kind to me but the patient load is making it very difficult for me to get treatment in the speed I want.”
B	“I go to the hospital whenever Appa goes. I cannot go alone.”
C	“Having kids to feed and a house to take care of, I cannot visit the hospital as I would like to. The doctors are nice and respectful but they cannot make all my problems disappear. I need money more than I need treatment.”

Subtheme# 3.2	Informal Support
A	“I wish I can pay back my family somehow. They did everything they could for me. My mother prepared warm milk for me. My father took money he knew he couldn’t pay back easily. My brother never said no when I asked them to take me to the hospital. I wish I hadn’t put them through all this. They even agreed to move out of the house for me.”
B	“The only not-so-horrible thing about this incident is that I have become closer to Appa.”
C	“My parents and kids have been extremely supportive. I cannot imagine what I would have done without them.”

## Discussion

The article explores the facts and figures related to acid burn attacks on the women and various factors associated with it. The number of acid burn victims is increasing in countries where rehabilitation services are the lowest [2]. These are the highest in Southeast Asia including countries like Bangladesh, Pakistan, India, China, Malaysia, Nigeria, Cambodia, and Uganda [7]. In Pakistan, these numbers are alarmingly high [1] and are considered now a topic worth discussion and research [4]. In Punjab, 48 out of 52 (92%) cases of violence against women were acid burn victims. This rate is alarmingly high, in comparison to Sindh where the rate is 3%. This shows that acid throwing is an issue of majorly populated areas, as depicted by our case study [3]. The reason for these high numbers is the easy and cheap availability of acids [5]. Acid violence against women is influenced by many factors like age, sex, ethnicity, social status, etc [7].

Data from Pakistan, particularly Punjab [8] suggests that psychological violence is much more than physical violence in Pakistan. One of the reasons behind this is the use of illegal arbitration systems like 'panchaits'(an illegal congregation of leaders and influential elders belonging to a rural area which decide punishments on matters of their area). Women fear going to the police or courts due to the concept of humiliation. And even when they contact the law, there is no legislation or proper policies to provide justice to acid burn victims. Our case indicates the same. The criminal was freed from jail because of the lack of proper laws.

Several factors have been associated with this acid throwing including the feeling of revenge, dowry issues, refusal of marriage issues, quarrels over land, and property [9]. In some cases, women’s higher financial status as compared to their spouse was associated with high rates of violence, specifically in post-Soviet countries [10]. Another factor linked to female violence was the concept of “wife-beating”. This culture is quite common in African and middle-east countries [11].

A study by García-Moreno emphasized the fact that the women who fall prey to partner violence also suffer from higher rates of different health-related issues and risk behavior including reproductive health problems, mental health problems, and deaths due to homicide or suicide [12]. A study conducted in Bangladesh states that acid violence often also affects small children sleeping beside the actual victim [7], which is similar to our case study. The same research states that acid burns can lead to social withdrawal and isolation, also seen in our study. Our case study poses similarities with a Cambodian case series that shows that acid burns have devastated and long-term medical effects, along with emotional deterioration [6]. Another research analyzed the emotional and social effects of acid burns according to Derriford and Rosenberg scales, and indicated depression due to physical appearance and low self-esteem [13]. Our victims also showed signs of depression and low self-esteem. A study shows that patients who experience physical or sexual assault such as acid throwing can suffer from post-traumatic stress disorder (PTSD). The symptoms of PTSD include nervousness, irritability, and the fear that the event might occur again [14]. Our patients showed all these signs.

A study by Patel states that a lot of formal and informal support is needed to rehabilitate acid burn victims [15]. Our victims had informal social support, but the lack of formal social is preventing them from proper rehabilitation. The same research also shows that social and cultural attitude towards acid burn victims is different in the eastern societies, leading to appearance-related distress. A study by Milton [16] also poses similarities to ours by stating that most victims are young females, who suffer from acid throwing by a dominant male, after refusing his proposal. Also, permanent facial scarring leads to social stigmatization. The social withdrawal and feeling of helplessness in our patients were aggravated by the lack of substantial laws to punish the wrong-doers. This is similar to Bangladeshi and Cambodian [17] cases, where the absence of legislation for acid burn victims is problematic for the patients. 

Social support is defined as the practical, emotional, and moral support provided to patients in formal and informal ways [18]. Low levels of social support have been found to have a qualitative relationship with post-traumatic stress disorder. Moreover, it has been reported that social support, both parental support and family conflict, can help in recovering from the traumatic incidents [19]. Social support helps in mental well-being as well as physical well-being because support increases levels of interleukin-1 that helps in wound healing [20]. Our patients depict similar findings. Informal social support has helped the patients recover and develop positive feelings, while more formal support is needed to enhance their healing process. Moreover, several gender training and equity programs are introduced which have resulted in the reduction of violence to 55% clearly showing positive results [21-22].

From the above discussion, we can say that researches from other countries share similarities with our case study. Many patients suffer from changes in life perspective, behavior, feelings, social withdrawal, social isolation, and depression. Formal and informal social support is vital for the proper and complete rehabilitation of acid burn victims. Many non-government organizations (NGOs) are working at the moment, but our public hospitals should be equipped with proper tools and services for rehabilitation. Moreover, to help with the recovery of patients and to prevent such accidents in the future, the government needs to formulate solid policies and rules. Enforcement of law and justice will work wonders in such situations. But data from Pakistan is not sufficient and more research is needed to convince the authorities to take proper action.

Limitations

Our study is limited by convenient sampling and a small sample size. Convenient sampling was adopted due to the lack of resources. The small sample size occurred largely due to the unwillingness of selected subjects to talk about the incident of vitriolage as it entailed an unpleasant recalling of their trauma.

Recommendations

We recommend the following steps to be taken, based on our experience while conducting this study:

1. Every government hospital, specially the tertiary ones, must have a separate, public rehabilitative center for patients who experienced vitriolage. These centers must work under the departments of plastic and reconstructive surgery. The management should be multi-disciplinary, with involvement of the departments of psychiatry/psychology. Every patient presenting to the hospital for initial evaluation/reconstruction/rehabilitation after an acid attack must be evaluated by a psychiatrist as well. This will ensure h improved quality of life, following trauma.

2. Hospitals dealing with acid burn patients must work with NGOs for providing social support. Formation of support groups should be encouraged, where victims of vitriolage meet to share and discuss the trauma they experienced. Departments dealing with such patients must have referral information for these support groups.

3. Multiple attempts have been made by the government to prevent acid and burn attacks. Punishments for such heinous crimes have also been decided. But all this legislation is on paper. Strict implementation of legislation via transparent work by police and judiciary is needed. Perpetrators of vitriolage must be tracked down and brought to justice on priority basis.

## Conclusions

Three patients suffering from vitriolage were studied in two sessions to analyze the change in life perspective after the incident and its effect on their social health. In conclusion, it can be said that the criminal act of acid-throwing causes physical damage as well as psychological damage. Surgery and medicine can deal with the scarring and the physical sequelae but complete rehabilitation must involve psychiatric and psychological therapy as well. The government should form separate public help centers for such patients. The need of the hour, however, is the invention and implementation of proper legislation for the prevention of this heinous crime.
